# Extracellular Vesicles in Haematological Disorders: A Friend or a Foe?

**DOI:** 10.3390/ijms231710118

**Published:** 2022-09-04

**Authors:** Ioanna Lazana

**Affiliations:** 1 Cell and Gene Therapy Laboratory, Biomedical Research Foundation of the Academy of Athens, 115 27 Athens, Greece; ilazana@doctors.org.uk; 2 Department of Haematology, King’s College Hospital NHS Foundation Trust, London SE5 9RS, UK

**Keywords:** extracellular vesicles, exosomes, haematological malignancies

## Abstract

Extracellular vesicles (EVs) have emerged as important mediators of homeostasis, immune modulation and intercellular communication. They are released by every cell of the human body and accordingly detected in a variety of body fluids. Interestingly, their expression can be upregulated under various conditions, such as stress, hypoxia, irradiation, inflammation, etc. Their cargo, which is variable and may include lipids, proteins, RNAs and DNA, reflects that of the parental cell, which offers a significant diagnostic potential to EVs. In line with this, an increasing number of studies have reported the important contribution of cancer-derived EVs in altering the tumour microenvironment and allowing for cancer progression and metastasis. As such, cancer-derived EVs may be used to monitor the development and progression of disease and to evaluate the potential response to treatment, which has generated much excitement in the field of oncology and particularly in haemato-oncology. Finally, EVs are able to transfer their cargo to target cells, modifying the properties of the recipient cell, which offers great therapeutic potential for EVs (either by specific drug delivery or by delivery of siRNAs and other inhibitory proteins). In this manuscript, we review the potential diagnostic use and therapeutic options of EVs in the context of haematological malignancies.

## 1. Introduction

Extracellular vesicles (EVs) consist of a lipid bilayer and have recently emerged as important mediators of homeostasis and cell-to-cell communication. Since their initial description, about 40 years ago, EVs have now been identified to be produced by almost all cell types in the human body and accordingly detected in a variety of fluid types, such as blood, breast milk, semen, cerebrospinal fluid, bronchoalveolar lavage and bile [1]. EVs, as natural products, participate in a number of normal processes, such as innate and acquired immunity, angiogenesis, tissue regeneration, cell differentiation, autophagy, coagulation, neural development, placental physiology and pregnancy [2]. Besides that, EVs also play an essential role in pathological conditions, and predominantly in neurodegenerative conditions and malignancy, and have attracted great scientific interest in recent years. More interestingly, their expression can be upregulated under various conditions, such as irradiation, hypoxia, acidosis, cell stress and cell activation [3], in order to facilitate the delivery of certain messages and generate a specific response to the aforementioned stimuli.

**Figure 1:**
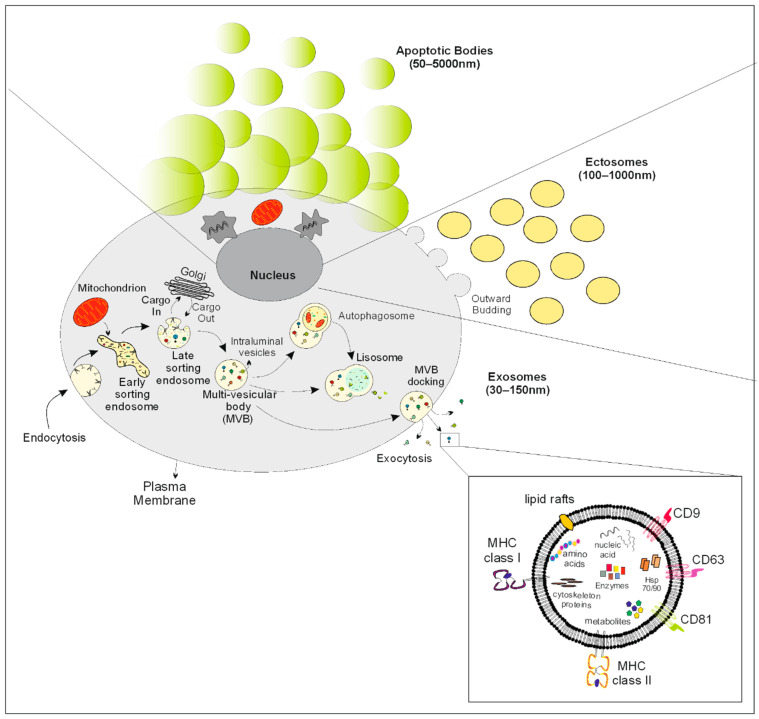
The figure illustrates the biogenesis, size and main features of the different classes of EVs.

EVs are quite a heterogeneous group of vesicles, both functionally and phenotypically. The International Society of EVs (ISEVs) has broadly categorised them into: (i) those derived from the plasma membrane (ectosomes, microvesicles and microparticles) and (ii) those derived from the endosomal system (exosomes) and specifically from the intraluminal vesicles (ILV) formed in the multivesicular body (MVB) (Figure 1) [4]. With regard to EV characterisation, the demonstration of the lipid bilayer is of imperative importance. Beyond that, several markers have been proposed to be associated with EV biogenesis (CD61, CD63, CD81, and TSG101, regarded as endosome-derived, and CD73, C1q, and ARRDC1, regarded as plasma membrane-derived), although their specificity remains questionable [5]. Size has also been used for EV categorisation, with exosomes constituting the smallest compartment. However, it is now evident that there is significant overlap in the size of different EVs, concluding that size alone cannot be used for EV categorisation.

The EV cargo is equally variable and may include proteins, lipids, RNAs (miRNA, lcg–RNA) and DNA. This cargo differs depending on its cellular source, reflecting that of the parental cell. More interestingly, EVs have the unique ability to transfer their content to target cells, modifying the biological properties of the recipient cell. This is mediated either by: (i) contact, referring to receptor–ligand interaction; (ii) uptake, referring to internalisation of the EVs; (iii) fusion, referring to fusion of EVs with the plasma membrane; or (iv) a combination of the above [6]. 

Overall, EVs have a number of unique properties that offer an advantage over cell and gene therapies, such as: (i) they lack a nucleus, so they cannot proliferate and/or differentiate in vivo; (ii) they have no metabolic potential, indicating that their functions cannot be altered by the host microenvironment; (iii) they are stable biological products and can be stored at −80 °C; and (iv) their small size allows them to circulate easily and reach distant places in microcirculation and also cross natural barriers (such as the blood–brain barrier). All the above are of great importance, as they offer an uncountable potential for therapeutic use. Indeed, there is a growing number of clinical trials involving the therapeutic use of EVs, as well as a number of companies exploring their diagnostic potential, with very encouraging results.

In this manuscript, we focus on the potential role of EVs in cancer. Specifically, we review the potential therapeutic use and diagnostic potential of EVs in the context of haematological malignancies (Table 1).

## 2. Haematological Malignancies

### 2.1. Myeloid Disorders

#### 2.1.1. Myelodysplastic Syndromes

Myelodysplastic syndromes (MDS) are a heterogeneous group of disorders, characterised by dysplasia, ineffective haematopoiesis and cytopenias and associated with an increased risk of progression to acute myeloid leukaemia (AML) [56]. Currently, the only available tool for risk stratification of patients is the Revised International Prognostic Scoring System (IPSS-R), which divides patients into very good, good, intermediate, poor and very poor prognoses [57], and the therapeutic approaches vary accordingly. However, the responses and survival outcomes vary significantly within the same IPSS-R patient groups receiving the same therapy, underlying the need for novel diagnostic tools, stratifying better the patients and predicting the responses to therapy.

Circulating small noncoding RNA (sncRNA) molecules, and particularly microRNAs (miRNAs), have attracted significant interest and various groups have identified either a unique miRNA signature or a combination of miRNA molecules to predict MDS outcomes [58,59]. Hrustincova et al. [7] were one of the first groups to explore the role of plasma EVs in the risk stratification of MDS. The authors reported a similar number of EVs in MDS plasma compared to healthy controls, but with significantly (almost 10-fold) higher sncRNA numbers. Similarly, a direct comparison between plasma and EV miRNA signature in patients with MDS revealed: (i) a significantly higher and more homogeneous RNA cargo in EVs, compared to plasma RNA content; (ii) different representation of miRNAs between plasma and EVs; and (iii) equally high levels of sncRNA in patients with early compared to advanced MDS, which is likely attributed to the excessive apoptosis noted in early stages of MDS. This indicates that plasma and EV RNA profiling represent different aspects of the same coin and one should not replace the other, but it should rather complement it for a better understanding of the disease. Finally, the authors identified a combined sncRNA panel for EVs that was able to predict patient outcomes. Those with the best predictive values were the miR-126-3p, miR-125a-5p, miR-199a-3p, miR-151a-3p and miR-423-5p. However, although these results strengthen the potential use of such EV miRNAs as noninvasive biomarkers in MDS prognostication, larger independent studies are required for validation prior to their inclusion in clinical practice.

Another study by Cerisoli et al. [8], comparing the phenotype and ‘inflammatory’ miRNA EV cargo from patients with low-risk MDS and healthy individuals, revealed a significantly higher EV CD34 expression in MDS patients, which did not correlate with blast percentage and did not predict disease progression. Downregulation of miR-16, miR-17, miR-20a, miR-21, miR-126, miR-181a, miR-146a and miR-155 was noted in MDS-derived EVs compared to those from healthy donors, suggesting their possible use as biomarkers of MDS development. However, follow-up studies are required to explore the kinetics of such miRNAs during disease progression, prior to their use as biomarkers of malignant transformation.

Enjeti et al. [60] investigated the potential contribution of EVs in the bleeding diathesis of MDS patients, as it is well known that a subset of MDS patients will experience significant bleeding, disproportionate to the level of the associated thrombocytopenia [9]. The authors demonstrated significantly lower procoagulant EV function in MDS patients compared to controls, as assessed by factor Xa and thrombin generation assays. However, the number of plasma EVs was similar in the two cohorts, failing to explain the difference in phospholipid content, although assessment by flow cytometry revealed a marked reduction in large EVs. A larger cohort of patients is required to confirm these findings and possibly examine bone marrow samples in addition to plasma, to gain more insight into abnormal megakaryopoiesis. Furthermore, miRNA-28, which is known to correlate with pulmonary embolism and thrombosis when raised, was found to be significantly reduced in MDS patients, suggesting a contribution to increased bleeding in these patients. Other EV miRNAs (such as miRNA-LET7D, -548J, -4485, etc.) were also found to be deregulated in MDS patients, but their significance was uncertain.

#### 2.1.2. Acute Myeloid Leukaemia

Since the first description of EV involvement in tumourigenesis by Skog et al. [61], there has been increasing evidence of the fundamental role of EVs in the pathogenesis of leukaemia. Fang et al. [10] were the first to describe markedly increased levels of miRNA-10b in serum EVs from patients with AML. It is well known that miRNA-10b suppresses myeloid differentiation and promotes AML cell proliferation and inhibition of apoptosis [62]. EVs have also been shown to support leukaemogenesis by affecting the microenvironmental niche. Specifically, it was demonstrated that AML cells secrete EVs, bearing important miRNAs, such as miR-150 and miR-155. These EVs traffic to haematopoietic stem cell and progenitor (HSPC) cells and suppress their proliferative and differentiation capacity, compromising haematopoiesis and supporting leukaemic persistence [11]. Along the same lines, Huan et al. [63] described the transfer of transcriptional factors and mRNA from AML to bystander (such as stromal and progenitor) cells via EVs, supporting leukaemia development, whereas Wojtuszkiewicz et al. [51] provided evidence of the EV-mediated release of antiapoptotic proteins (such as MCL-1, BCL-2 and BCL-XL) and uptake by myeloid blasts, inducing apoptosis resistance.

A number of studies also describe the role of EV-derived miRNAs in supporting the survival of leukaemic cells in the bone marrow (BM) microenvironment. For example, EV-derived miR-125b has been shown to inhibit the expression of proapoptotic P53, BAK and Bmf and promote cell cycle [64], whereas EV-derived miR-26a-5p facilitates AML development through activation of Wnt/B-catenin signalling pathway [12]. Beyond miRNAs, other EV-derived long noncoding RNAs (lnc-RNA) have been shown to contribute to the generation of a leukaemia-permissive microenvironment, such as EV-circ-0009910, which regulates miR-5195-3p and growth factor receptor-bound protein 10 (GRB10), promoting cell cycle progression [13] and EV-circ-0004136, which promoted AML cell viability, migration and invasion via miR-570-3p, which modulates TSPAN3 expression, allowing an autonomous proliferation of AML cells [14]. Other EV cargo, supporting leukaemogenesis, includes DKK1, which is uptaken by BM stromal cells leading to inhibition of osteogenesis and upregulation of haematopoietic stem cell-supporting factors (such as CXCL12, KITL and IGF1) [15], and BMP-2, which transfers to mesenchymal stem cells (MSCs) and upregulates connective tissue growth factor (CTGF) promoting osteogenic differentiation through Smad1/5 signalling [65]. Finally, EVs play an important role in leukaemia cell survival by promoting angiogenesis, either directly, by delivering IL-8 and vascular endothelial growth factor (VEGF) changing the proliferative capacity of endothelial cells [66] and delivering miR-17-192, which stimulates angiogenesis [16], or indirectly by inducing hypoxia, through delivery of hypoxia-inducible factor 1-a (HIF-1a) to endothelial cells, which respond by activation of their angiogenic properties [67].

Beyond the contribution of EVs in the development and survival of AML blasts, emerging data also support their involvement in conferring drug resistance. Viola et al. [34] were one of the first to provide evidence that EVs confer chemoresistance to AML cells. Specifically, they demonstrated that EVs transfer transforming growth factor-beta (TGF-β) and miR-155 and miR-375 from leukaemia cells to MSCs, leading to stromal cell protection of AML cells. Along the same lines, miR-125b was found to be upregulated in drug-resistant acute promyelocytic leukaemia (APML) cells, whereas transfection of AML cells with miR-125b rendered them resistant to chemotherapeutic drugs [35]. Furthermore, Bouvy et al. [36] demonstrated a direct transfer of the multidrug resistance protein 1 (MRP-1) between daunorubicin-resistant AML cells and other leukaemic cells, whereas Hong et al. [68] suggested that AML-derived EVs could confer resistance to adoptive cell therapies. Specifically, they demonstrated that EVs derived from plasma of AML patients treated with NK-cell therapy, when given to nontreated AML cells protected them from NK-cell lysis. Interestingly, EVs were not internalised by AML cells, but they conveyed the resistance by delivering inhibitory signals to recipient cells, such as the TGF-β inhibitory signalling.

Finally, EVs have been proposed to be a useful diagnostic tool for disease diagnosis, minimal residual disease monitoring and risk stratification [69]. For example, significantly higher levels of plasma EV miR125 expression at diagnosis were shown to correlate with an aggressive course, with a higher risk of relapse and significantly shorter survival rates in intermediate-risk AML patients [39]. However, no longitudinal analysis of EV-miR125 levels was performed, comparing the values before and after each cycle or type of treatment to determine whether they could assist in deciding which patient would benefit from treatment continuation or change. Furthermore, it would be interesting to see whether EV-miR125 levels could be used either alone or in combination with the currently used markers to better stratify AML patients. EV-miR-10b has also been proposed to be an independent prognostic marker in patients with AML disease. Specifically, it was demonstrated that EV miRNA-10b levels were associated with significantly shorter overall survival (OS) and disease-free survival (DFS) [10]. However, the small cohort size and the failure to subtype AML patients limit the translational potential of this marker. Other EV miRNAs, such as miR-1246 [40], miR-532 [41] and miR-125b [39], have also been suggested to predict relapse or poor outcomes, providing a valuable perspective on the disease course. Kontopoulou et al. evaluated the diagnostic potential of plasma EVs, by performing next-generation sequencing (NGS) and fragment-length analysis in plasma EV-derived DNA from paediatric patients with AML disease [42]. The authors demonstrated an absolute mutational and single nucleotide polymorphism (SNP) correspondence between EV and plasma DNA, suggesting that EV-DNA analysis could complement AML diagnostics and potentially treatment responses. However, larger cohort studies are required to validate the results and facilitate their clinical translation.

#### 2.1.3. Myeloproliferative Disorders

The involvement of EVs in pathogenesis, disease diagnosis and monitoring of myeloproliferative disorders (MPDs) has been well described.

In the context of chronic myeloid leukaemia (CML), Jafarzadeh et al. demonstrated an altered T-cell function upon exposure to K562 cell-derived EVs [17]. Specifically, an increase in expression of immunosuppressive molecules, such as IL-10, IL-6, IL-17, nitric oxide (NO) and FOXP3, along with a concurrent suppression of T-cell activation markers, such as CD3d and NFATc3 was shown, suggesting that CML-derived EVs are able to modify immune cells to facilitate tumour survival. In these lines, Zhang et al., suggested a proleukaemic effect of CML-derived EVs on haematopoietic stem cells (HSCs), by demonstrating a dysregulation in certain transcriptional factors and miRNAs (such as miR-146b-5p), associated with leukaemia transformation [18]. With regard to the role of EVs in CML pathophysiology, it has been demonstrated that the BCR-ABL gene can be transferred to normal neutrophils altering their functions, via CML-derived EVs [43]. More importantly, CML-derived EVs were proven to cause CML disease in an NSG mouse model, underlying the contribution of EVs in tumourigenesis and tumour progression [44]. Of course, further work is required to confirm these findings, as the authors failed to fully characterise CML-derived EVs and possibly explore other genes transferred along BCR-ABL. In terms of pathogenesis, the involvement of CML-EVs in angiogenesis, which constitutes an essential part of tumourigenesis, has been well described [45]. There is evidence suggesting that CML-EVs: (i) transfer miR-92a and activate src signalling [19]; (ii) promote tube formation in endothelial cells, via upregulation of miR-210 [20]; and (iii) affect vascular remodelling via IL-8-mediated alteration of VCAM-1 [21]. Caivano et al. [46] reported an increased expression of CD13 in serum EVs from patients with CML disease, compared to healthy donors. However, the authors failed to characterise CML-EVs further, either phenotypically or transcriptionally. The potential use of CML-derived EVs as a potential biomarker has also been explored. Kang et al. [70] were the first to identify the presence of the 250 bp BCR-ABL transcript in EVs derived from CML human cell lines and from patients with CML disease. In the latter, the EVs were derived from the bone marrow serum and the BCR-ABL protein was detected only in patients in the blast and accelerated phases. Interestingly, this disappeared after treatment with tyrosine kinase inhibitors (TKI), which correlated nicely with the clinical response. However, the study has several limitations, such as: (i) the very small cohort size, with only three patients included; (ii) a commercially available kit (ExoQuick) was used to isolate EVs, which comes with its own limitations; and (iii) inability to interpret bands with weaker intensities identified in the chronic phase. On the other hand, Bernardi et al. [71] used the peripheral blood (PB) of patients in the chronic phase (CP) of the disease, who were also receiving TKI treatment, as an EV source. A total of 10 patients in CP and in deep molecular remission (DMR) were used. Results confirmed both a tumour-EV enrichment in PB of CML patients by immune-affinity methods and the detectability of the BCR-ABL transcript in such EVs.

The contribution of EVs in the pathogenesis of thrombotic complications in patients with BCR-ABL1 negative MPDs has been suggested by various groups [72]. Significantly increased levels of EVs were noted in the plasma of patients compared to healthy individuals. Moreover, the increased EV levels were positively correlated with the presence of thrombotic risk factors, whereas a decrease both in the EV levels and the thrombotic events was noticed with the application of cytoreductive therapy, implying an association between the two [73]. However, the source of EVs was not defined, and neither was the presence of other contributing procoagulant factors. Furthermore, the involvement of other larger particles could not be excluded, based on the isolation method. Along the same lines, Taniguchi et al. [47] demonstrated a significant association between tissue factor (TF)-expressing EVs and thrombotic events in patients with MPDs, suggesting a possible prognostic role of EVs for thrombotic complications, although in a small study. Larger prospective studies would be required to validate this result in order to be used for risk stratification of thrombotic risk. Ahadon et al. [74] also reported significantly increased levels of plasma EVs of platelet origin in patients with polycythaemia vera (PV), although the associated thrombotic and other complications of the disease were not sought. Another interesting study by Poisson et al. [75] used erythrocyte-derived microparticles from patients with JAK2V617F MPDs that were able to promote NO signalling and exert increased responses to vasoconstrictors, both in vitro and in vivo on femoral arteries. Subsequent proteomic studies proposed increased myeloperoxidase expression as the causative factor, with antioxidants reversing the effect of excess vasoconstriction.

Similarly, significantly increased EV plasma levels, of platelet and endothelial origin, were detected in patients with essential thrombocythaemia (ET) [48]. Further analysis revealed an association with elevated von Willebrand factor levels and thrombin generation, suggesting that EVs participate in this hypercoagulable state that characterises patients with ET. This is further supported by the positive correlation observed between the International Prognostic Score of Thrombosis (IPSET) score and the degree increase in EV levels. Whether the increased EV levels drive the hypercoagulability or rise versa, remains to be defined. Similar results have been observed by other groups [76], along with a normalisation of the EVs to levels comparable to those of healthy individuals [77].

In line with the above, significantly elevated levels of platelet-derived EVs and decreased levels of megakaryocyte-derived EVs were detected in patients with myelofibrosis (MF) [78]. More interestingly, the EV levels normalised upon treatment with ruxolitinib, and the degree of improvement was proven to be able to discriminate between responders and nonresponders, in regard to the spleen size, emphasising the potential role of EVs as a useful response biomarker. A study by Forte et al. [22] demonstrated an essential role for plasma EVs in promoting survival and migration of CD34+ cells in triple-negative (TN) MF patients. More, specifically, the authors detected a unique phenotype of plasma EVs, along with a unique miRNA signature, with upregulation of miR-34a-5p, -127-3p, -212-3p and miR-361, promoting an ‘MF-susceptible’ microenvironment.

### 2.2. Lymphoid Disorders

#### 2.2.1. Acute Lymphoblastic Leukaemia

A role for plasma EVs in the pathogenesis of acute lymphoblastic leukaemia (ALL) has been proposed by Yan et al. [23]. The authors demonstrated a significant upregulation of miR-181b-5p in EVs, which were able to be internalised by ALL cells and promote their survival, migration and invasion. However, the authors failed to identify the cellular source of such EVs or the expression of other miRNAs carried by the EVs. Furthermore, although the uptake of miR-181b-5p-carrying EVs by ALL cells was demonstrated, their internalisation was not proven. Finally, it would have been interesting to have inhibitory studies, ‘blocking’ the miR-181b-5p expression, as a means to confirm their role in ALL cell proliferation and survival. Another study suggested that ALL-derived EVs can promote the growth of cells with low/no-proliferative capacity, promoting their survival [79]. However, no mechanism of action or possible contributing factors were suggested by the authors, so further studies are required to confirm/validate these results. An elegant study by Haque et al. [24] also supported the role of EVs in the pathogenesis of ALL. The authors demonstrated that plasma EVs from paediatric ALL patients promote leukaemic cell proliferation and survival by altering gene expression profiles (i.e., upregulating essential for survival genes, such as BCL-2 and MCL-1 and downregulating proapoptotic genes, such as BAX). More interestingly, miR-181a was proven to play a key role in the aforementioned effects, as its silencing led to inhibition of leukaemic cell proliferation, providing evidence of a novel therapeutic target in the treatment of ALL.

With regard to the prognostic role of EVs in ALL, Egyed et al. [80] identified for the first time a unique population of EVs in the cerebrospinal fluid (CSF) of patients with central nervous system (CNS)-positive disease. These unique EVs, characterised by the absence of the characteristic markers CD63 and CD81 and low-end size range, were excessively high in patients with CNS+ disease. Although these results are very exciting, the low patient number limits their clinical translation, necessitating larger studies for verification.

A very interesting study by Fei et al. [37] demonstrated the potential use of EVs in conferring drug resistance. The role of galectin-3 in the protection of ALL cells that are under the stress of drug therapy is well established, although its origins remain elusive [81]. The authors in this study detected stromal fibroblasts as a major source of galectin-3, which was able to protect ALL cells from chemotherapy. More interestingly, EVs derived from these stromal cells were also found to carry galectin-3, whereas neither plasma EVs, nor pre-B ALL cell-derived EVs, seem to contain galectin-3, suggesting that this pathway could be targeted to overcome the protective effects of the stroma.

#### 2.2.2. Chronic Lymphocytic Leukaemia

EVs have been demonstrated to play a crucial role in the pathogenesis of chronic lymphocytic leukaemia (CLL). An elegant study by Comprot et al. [82] revealed that EVs derived from CLL-BM-MSCs offer the following survival advantages to leukaemic B cells: (i) improve their migratory capacity, so they can ‘hide’ into the stromal niches and get protected from chemotherapeutic drugs; (ii) rescue B cells from spontaneous apoptosis; and (iii) increase chemoresistance against eight different drugs through BCR-pathway activation. These results provide evidence of the essential role of EVs in generating/protecting the leukaemic microenvironment (ME), suggesting that this interaction may be a new target for therapeutic interventions. However, the authors studied only the impact of BM-MSCs-derived EVs on leukaemic B cells, but it would be very interesting to elucidate the role of EVs derived from other cells (such as T cells, antigen-presenting cells, natural-killer cells, etc.) and their impact on the tumour microenvironment. Another study by Smallwood et al. [25] elucidated the role of tumour (CLL)-derived EVs in promoting ME modification and immune evasion and angiogenesis, by studying the impact of such EVs on CD4+ T cells. Results revealed that CLL-derived EVs: (i) contain miR-363 that targets the immunomodulatory molecule CD69 and (ii) alter the function of CD4+ T cells, promoting their proliferation, migration and immune synapse signalling, modifying the communication with the ME. However, the contribution of other miRNAs into the crosstalk between CLL-derived EVs and immune cells, along with the impact of other local ME factors (such as hypoxia, hypoglycaemia, cytokine milieu, etc.) was not addressed in the current study.

With regard to the potential prognostic role of EVs in CLL, there are various studies supporting its utility for prognostication and minimal residual disease (MRD) [83]. It is now evident that CLL patients express significantly higher numbers of EVs and CD19-expressing EVs, which decrease significantly upon treatment with Bruton’s tyrosine kinase (BTK) or phosphoinositide 3-kinase (PI3K) inhibitors. Furthermore, a significant correlation has been described between serum EV levels and RAI staging, whereas no such correlation was detected with the level of somatic hypermutation. However, when CLL cells were stimulated for 24 h in vitro, a significant upregulation of EVs was noted, which correlated nicely with the mutational status of patients. Finally, plasma EVs and particularly CD19-expressing EVs were not correlated with the absolute lymphocyte count, supporting the notion that EVs reflect better the disease status compared to plasma, since CLL cells reside within the lymphoid system.

EVs have also been suggested to serve as important tools for the development of novel therapeutic strategies. Gartner et al. [52] manipulated EVs to circumvent the T-cell anergy that characterises CLL disease. Specifically, the authors engineered EVs to carry: (i) the glycoprotein gp350, which not only acts as a neo-antigen for CD4+ T cells but also promotes B-cell tropism; (ii) the CD40 ligand, enhancing costimulation and thus immunogenicity; and (iii) the herpesviral protein pp65, which mediates a strong immune (both CD4+ and CD8+) response. CLL cells, upon uptake of these EVs, exhibited potent antigen-presenting capacity (specific for gp350 and pp65), inducing the reactivation of specific cytotoxic T cells (against gp350 and pp65). More interestingly, these target-specific cytotoxic T cells could be selectively expanded upon coculture with EV-treated CLL cells, potentiating their translational capacity. On the other hand, it has been demonstrated that EVs, produced by tumour cells, facilitate immune escape and even inhibit adoptive cell immunotherapies, such as Chimeric Antigen Receptor (CAR)-T-cell therapy. Specifically, it was shown that antiCD19 CAR-T cells (CART19) alter their phenotype, expressing inhibitory receptors (such as CTLA-4) and become anergic and exhausted, upon coculture with CLL-derived EVs [84], exhibiting an impaired antitumour activity. Although the exact mechanism by which CLL-derived EVs inhibit CAR-T-cell function remains unknown, these results highlight that EVs may be important therapeutic targets for successful immunotherapy.

#### 2.2.3. Lymphomas

An important contribution of EVs in pathogenesis, prognostication and disease response of lymphomas has been suggested by several studies [85]. An elegant study by Koch et al. [86] provided evidence of the important contribution of the EVs in tumour cell communication, equilibration and progression. The authors reported that side population (SP) cells and non-SP cells are able to exchange information, leading to altered function, through EVs. More specifically, exchanged Wnt signals between SP and non-SP cells, through EVs, allow the conversion of non-SP cells back to SP phenotype, offering them a clonogenic potential. This is of great importance as it offers a clear target for intervention, preventing tumour progression. Another study on Hodgkin lymphoma (HL), demonstrated the role of EVs in supporting tumour growth [26]. HL-derived EVs were proven to be uptaken by stromal fibroblasts and alter both their secretome, releasing proinflammatory and proangiogenic cytokines and their signalling pathways, promoting TNF-a/NFkB signalling. Overall, this interaction supports a favourable ME for tumour growth and survival, although identifying the molecular pathways of EV activity would offer a greatly better understanding of the pathophysiology of HL.

Diffuse large B-cell lymphoma (DLBCL)-derived EVs have been shown to differentiate between different subtypes (germinal centre B-cell GCB and activated B-cell ABC) and to express a unique pattern of proteins involved in B-cell receptor (BCR) and ErbB signalling and Fc-gamma-mediated phagocytosis and NK-mediated cytotoxicity [87]. More interestingly, these components were shown to be significantly enriched in EVs, compared to plasma from DLBCL patients, suggesting that important elements of immune responses are preferentially packaged into EVs. Another study by the Spanish Lymphoma Oncology Group investigated the possible prognostic role of mRNAs in plasma EVs from 60 patients with DLBCL and 38 with follicular lymphoma (FL) [27]. Interestingly, a significantly elevated expression of BCL-6 was detected in patient EVs, compared to healthy controls, whereas BCL-6 and c-Myc mRNA levels were shown to predict shorter OS and worse progression-free survival (PFS) in FL patients. Furthermore, a reverse association was noted between the presence of AKT mRNA in EVs and response to rituximab. Although very encouraging these results in identifying high-risk and nonresponder patients, larger studies are required to confirm their validity. Similar studies in Hodgkin lymphoma patients, demonstrated that plasma EV-mRNA repertoire is predictive of responses [38]. Specifically, significantly elevated levels of specific mRNAs (miR24-3p, miR127-3p, miR21-5p, miR155-5p, let7a-5p) were detected in EVs from plasma of untreated patients, compared to controls. More interestingly a decline in these levels was detected upon treatment, which corresponded to metabolic response, based on PET-CT findings, whereas a further increase noted again in relapsed patients. However, larger prospective studies would be required to validate these results and incorporate the predictive potential of EVs into clinical practice. Along the same lines, Feng et al. [88] identified two miRNAs (miR-99a-5p and miR-125b-5p) that were enriched in plasma EVs and were able to predict chemotherapy resistance and subsequently poorer outcomes. Another study reported that higher levels of plasma CD9+/CD63+ and PD-L1+/CD63+ EVs correlated with adverse clinical features, therapeutic failure and poor outcomes in patients with DLBCL [49]. Interestingly, PD-L1+/CD63+ EVs were detected in all DLBCL patients, as opposed to cellular PD-L1 expression, which is only detected in about 20% of patients with DLBCL disease. Further to these results, it would be interesting to explore the possible predictive role of plasma PD-L1+/CD63+ EVs in patients receiving immune checkpoint inhibitors. Furthermore, extensive characterisation of such EVs, either phenotypically and/or molecularly, might offer great predictive potential.

With regard to the potential therapeutic use of EVs in the context of lymphoma, Chen et al. [53] demonstrated that tumour-derived EVs (termed TEX), carrying tumour-associated antigens (TAA), can be uptaken by host dendritic cells (DCs), without affecting their antigen-presenting capacity or viability, as opposed to T cells, which become anergic upon EV uptake. DCs were subsequently shown to present TAAs, enhancing the antitumour immune responses in vivo, in a lymphoma mouse model. These results offer a novel source of TAAs for targeted vaccine therapy, although standardisation of EV isolation and characterisation would be required prior to clinical translation.

#### 2.2.4. Multiple Myeloma

Emerging data support the role of EVs in generating a favourable ME promoting the survival and growth of multiple myeloma (MM) cells [89]. It has been demonstrated that MM-derived EVs promote angiogenesis and endothelial cell growth and create an immunosuppressive ME via upregulation of immunosuppressive molecules (such as inducible nitric oxide synthase-iNOS) and fostering of myeloid-derived suppressor cells (MDSCs) [28]. Interestingly, in vivo administration of MM-derived EVs in MM naive mice led to the generation and expansion of MDSCs, with potent immunosuppressive properties, through the STAT3 pathway, offering a novel therapeutic target. Another study demonstrated that under the chronic hypoxic conditions of the BM, MM cells secrete EVs containing miR-135b, which are subsequently uptaken by stromal endothelial cells [29]. As a result, there is an upregulation of the hypoxia-inducible factor (HIF)-1, which promotes angiogenesis. However, further work is required to fully characterise all the miRNAs contained in the EVs and how these, along with other soluble factors present in the MM ME, affect their function. EVs have also been suggested to contribute to osteogenesis suppression in various ways. Li et al. [30] demonstrated that MM-derived EVs, containing IncRUNX2-AS1, a natural antisense transcript, are uptaken by stromal MSCs. This leads to suppression of the RUNX-2 mRNA in MSCs, resulting in impaired osteogenesis. Furthermore, inhibition of EV formation, by administration of GW4869 in mice, led to amelioration of lytic bone lesions, confirming the pivotal role of EVs in the crosstalk between MM and stromal cells. Another study by Raimondo et al. [31] explored the contribution of MM-derived EVs in osteoclastogenesis. The authors demonstrated that the epidermal growth factor receptor (EGFR) ligand amphiregulin (AREG), which is related to increased bone resorption, is abundantly present in EVs from MM cells. Furthermore, treatment of MSCs with these AREG-containing EVs, resulted in release of IL-8, which is known to be associated with increased osteolysis, through osteoclast (OC) formation. This is of importance, as EGFR inhibitors could be deployed to inhibit the crosstalk between MM-derived EVs and MSCs in an effort to inhibit OC formation and bone destruction. Along the same lines, MM-derived EVs have been shown to promote osteoclast migration, differentiation and survival, through expression of markers such as CXCR4, RANKL, CTSK, MMP9 and TRAP and activation of the AKT pathway [32], contributing further to the understanding of the lytic bone disease pathogenesis in MM.

EVs have proven to be a powerful tool in predicting disease progression and poor outcomes in various malignancies. In the context of MM, Manier et al. [33], investigated the potential prognostic significance of small RNAs packed in MM-derived EVs. Results revealed that the miRNAs are the most abundant small RNAs in EVs and that two of them, let-7b and miR-18a were able to predict the outcomes (PFS and OS). More importantly, these markers maintained their prognostic capacity even after adjusting for adverse cytogenetics and the international staging system. Although these results are very promising, further validation in larger prospective studies is required to confirm the association between MM-derived EVs and outcomes in patients with newly diagnosed MM. Similarly, Iaccino et al. [50] reported that using idiotype (Id)-peptides (that bind the immunoglobulins expressed by malignant B cells) to detect MM-derived EVs they were able to predict disease progression, and most interestingly prior to biochemical progression (such as paraprotein rise). This novel platform of EV isolation based on fluorescent-labelled Id-peptides appears to be very promising in detection of minimal residual disease and early progression and has potential applicability in every malignancy, but further testing is required to confirm its validity, consistency and cost-effectiveness.

With regard to treatment of the disease, EVs have been explored both as potential vehicles of therapeutic cargo and as potential targets for a successful adoptive cell therapy. Garg et al. [90] demonstrated that MM-derived EVs significantly reduced the in vitro cytotoxic activity of NK- and active expanded NK cells, in a time- and dose-dependent way. However, the contribution of other soluble factors was also shown to be important in the inhibition of NK-cell function and probably a synergistic effect of the above might be responsible for the in vivo failure of such therapies. This remains to be defined in future prospective studies. On the other hand, MM-derived EVs have also been proven to have a favourable role when treating MM. Specifically, Borrelli et al. [54] demonstrated that treatment with drugs such as melphalan and doxorubicin promotes the production of EVs by senescent MM cells. Interestingly, these EVs were shown to carry the IL15/IL15RA complex, which subsequently triggers the activation and proliferation of NK cells promoting an antitumour immune response. However, an extensive characterisation of such EVs would be necessary to define the precise effect of senescence on tumour progression or repression. EVs have also been implicated in drug resistance in MM. Wang et al. [55] demonstrated that bone marrow stromal cells (BMSCs) produce EVs that promote the survival, proliferation and migration of MM cells. through multiple chemotactic- and angiogenesis-related proteins. Furthermore, in the presence of chemotherapeutics, such as bortezomib, the BMSC-derived EVs protected the MM cells from lysis in a dose-dependent way. Exploring the mechanism of such protection, the authors noted that MSC-derived EVs were able to reverse the Bortezomib-mediated bcl-2 inhibition and inhibit the cleavage of caspase-3 caspase-9 and PARP, offering a survival advantage to MM cells. Of course, other molecular pathways relating to drug resistance and cell survival need to be explored, in association with the EVs. Furthermore, the interplay between EVs, derived from various stromal cells and MM cells, carrying different cargos, needs to be further investigated.

## 3. Conclusions

With the emergence of new concepts relating to the involvement of EVs in the development and progression of malignancies, but also in their capacity to deliver their cargo to target cells, much excitement has been generated in the field of haematological malignancies. In this review, we presented and critically reviewed the most important work relating to EVs in the context of pathogenesis, diagnosis and therapeutics of haematological malignancies. More specifically, it is now well established that EVs support the survival of cancer cells, either by altering the microenvironment or by inducing apoptosis resistance and stimulating angiogenesis. Furthermore, EVs contribute to the development and survival of malignant cells by conferring drug resistance via multiple mechanisms. Besides the above, there is increasing evidence that EVs constitute a useful diagnostic tool for disease diagnosis, minimal residual disease monitoring and risk stratification, conferring valuable information on the disease course and outcomes. Finally, EVs have been suggested to serve as important tools for the development of novel therapeutic strategies, owing to their capacity to carry proteins, RNAs and DNA and to deliver their cargo to target cells, altering their function. The unique properties of EVs, such as the lack of nucleus and their very small size, constitute them superior candidates for successful immunotherapy, compared to currently used cell and gene therapies. All the above underline the particularly important contribution of EVs in cancer diagnostics and therapeutics and open a new horizon for: (i) early, minimally invasive diagnostics; (ii) personalised therapies with minimal/no side effects; and (iii) prompt detection of disease relapse or resistance. Furthermore, with the increasing awareness of the EV contribution to cancer biology, the prognostication and risk assessment for most haematological malignancies (such as acute and chronic leukaemias, myelodysplastic syndromes, multiple myeloma, etc.) may be improved, changing patients’ outcomes for the better.

However, and despite the increasing data supporting the important contribution of EVs in diagnostic, prognostic and therapeutic strategies in haematological malignancies, there are still some obstacles to overcome prior to their clinical translation and large-scale production. These include: (i) absence of a standardised protocol for EV isolation (various techniques have been employed for EV isolation, such as ultracentrifugation, ultrafiltration, size-exclusion chromatography, tangential-flow filtration, magnetic separation, etc., each with their own advantages and disadvantages); (ii) absence of a unique marker for EV detection and characterisation; (iii) heterogeneity in EV function, which is further affected by the isolation method employed, type and culture conditions of the cell source; and (iv) the regulatory requirements for clinical-grade production remain unclear [91]. The increasing awareness, interest and excitement on EVs from the haematology–oncology (and not only) community, along with the constant development of novel isolation and characterisation techniques continues to create novel means to overcome limitations and allow for further clinical translation, with the utter aim of better patient care and clinical outcomes.

## Figures and Tables

**Table 1:** **Overview of diagnostic, prognostic and therapeutic potential of EVs in the context of haematological malignancies.** The table illustrates the contribution of EVs to haematological malignancies and more specifically in their role as a biomarker, in disease outcomes, in drug resistance and as a therapeutic target. Furthermore, the associated EV cargo and resulting effect are demonstrated.

EV Role	Target	Effect	Haematological Malignancy	Ref.
**Biomarker**	Upregulated miR-126-3p, miR-125a-5p, miR-199a-3p, miR-151a-3p and miR-423-5p,	-	MDS	[7]
	Downregulated miR-16, miR-17, miR-20a, miR-21, miR-126, miR-181a, miR-146a, and miR-155	-	MDS	[8]
	Downregulated miR-28	Increased bleeding	MDS	[9]
	miR-10b	Suppressed myeloid differentiation and inhibited apoptosis	AML	[10]
	miR-150 and miR-155	Suppressed proliferative and differentiation capacity of HSPC	AML	[11]
	miR-125b, miR-26a-5p	inhibited the Expression of proapoptotic molecules	AML	[12]
	lnc-RNA, such as circ-0009910 and circ-0004136	Promote cell cycle progression and AML cell viability and invasion	AML	[13,14]
	DKK-1	Inhibited osteogenesis	AML	[15]
	IL-8, VEGF	Stimulated angiogenesis	AML	[16]
	IL-10, IL-6, IL-17, NO, FOXP3	Facilitated tumour survival	CML	[17]
	Dysregulation of miR-146b-5p	Associated with leukaemia transformation	CML	[18]
	miR-92a, miR-210, IL-8	Promoted angiogenesis	CML	[19,20,21]
	miR-34a-5p, -miR-127-3p, miR-212-3p, miR-361	Promoted survival and migration of CD34+ cells	MF	[22]
	Upregulation of miR-181b-5p	Promoted survival, migration and invasion of ALL cells	ALL	[23]
	Upregulation of survival genes and downregulation of proapoptotic genes via miR-181a	Leukaemic proliferation and survival	ALL	[24]
	miR-363	Altered the function of CD4+ T cells	CLL	[25]
	Promote TNF-a/NFkB signalling	Altered ME to support tumour growth and survival	HL	[26]
	Elevated BCL-6 expression	-	DLBCL	[27]
	Upregulation of I/S molecules (such as iNOS)	Promote MDSCs and create I/S ME	MM	[28,29]
	miR-135b	Upregulation of HIF-1, which promoted angiogenesis	MM	[30]
	IncRUNX2-AS1	Osteogenesis suppression	MM	[31]
	Increased AREG	Increased bone resorption	MM	[32]
	Increased CXCR4, RANKL, CTSK, MMP9, TRAP	Promoted osteoclast migration, differentiation, and survival	MM	[33]
**Drug resistance**	TGF-β, miR-155, miR-375	Stromal protection of AML cells	AML	[34]
	miR-125b	-	APML	[35]
	MRP-1	-	AML	[36]
	Galectin-3	Protective effect of stromal fibroblasts on ALL cells	ALL	[37]
	Increased AKT mRNA	Reduced response to rituximab	FL	[27]
	miR-99a-5p and miR-125b-5p	Chemotherapy resistance and poor outcomes	HL	[38]
**Disease outcomes**	Increased miR-125	Aggressive course, higher risk of relapse and significantly shorter survival	AML	[39]
	Increased miR-10b	Significantly shorter OS and DFS	AML	[10]
	Increased miR-1246, miR-532 and miR-125b	Predicted for relapse and poor outcomes	AML	[17,18,19,20,21,39,40,41,42,43,44,45,46]
	Elevated TF	Thrombotic events	MPD	[47]
	Elevated vWF and thrombin	Hypercoagulable state	ET	[48]
	BCL-6 and c-myc mRNA	Predict shorter OS and worse PFS	FL	[27]
	High levels of CD9/CD63 and PD-L1/CD63 EVs	Correlate with therapeutic failure and poor outcomes	DLBCL	[49]
	let-7b and miR-18a	Predicted for worse OS and PFS	MM	[50]
**Target**	Antiapoptotic proteins (such as MCL-1, BCL-2 and BCL-XL)	Induction of apoptosis resistance	AML	[51]
	Silencing of miR-181a	Inhibition of leukaemic cell proliferation	ALL	[24]
	EVs carrying gp350, CD40 and pp65	Exhibited strong Ag-presenting capacity and induces specific cytotoxic T cells	CLL	[52]
	Exchanged Wnt signals between SP and non-SP cells	Allowed tumour cell communication and progression	HL	[26]
	Vaccine using TEX-carrying TAA	Enhanced antitumour responses in mouse models	Lymphoma	[53]
	Inhibition of EV formation	Amelioration of lytic lesions in mice	MM	[31]
	Melphalan- and doxorubicin-induced EVs	Carried IL15/IL15RA, which promotes an antitumour response	MM	[54]
	MSC-derived EVs	Reversed the bortezomib-induced bcl-2 inhibition and inhibit the cleavage of cas3, cas9 and PARP	MM	[55]

**Abbreviations**: MDS: myelodysplastic syndrome, AML: acute myeloid leukaemia, HSPC: haematopoietic stem cell and progenitor cells, lnc-RNA: long noncoding RNA, DKK-1: dickkopf-related protein 1., VEGF: vascular endothelial growth factor, TGF-β: transforming growth factor-β, MRP-1: multidrug-resistant protein-1, OS: overall survival, DFS: disease-free survival, NO: nitric oxide, MPD: myeloproliferative disorders, TF: tissue factor, vWF: von Willebrand factor, ET: essential thrombocythaemia, MF: myelofibrosis, ALL: acute lymphoblastic leukaemia, CLL: chronic lymphocytic leukaemia, SP: side population, HL: Hodgkin lymphoma, TNF-a: tumour necrosis factor-a, ME: microenvironment, DLBCL: diffuse large B-cell lymphoma, FL: follicular lymphoma, PFS: progression-free survival, TEX: tumour-derived EVs, TAA: tumour-associated antigens, I/S: immunosuppressive, iNOS: inducible nitric oxide synthetase, MDSCs: myeloid-derived suppressor cells, MM: multiple myeloma, HIF-1: hypoxia-inducible factor 1, AREG: amphiregulin, cas3: caspase 3, PARP: poly (ADP-ribose) polymerase.
